# Interest in cardiothoracic surgery: are women more interested today?

**DOI:** 10.1007/s12055-025-02017-3

**Published:** 2025-08-13

**Authors:** Ana Lopez-Marco, Ralitsa Baranowski, Indu Deglurkar

**Affiliations:** 1 Department of Cardiac Surgery, St Bartholomew’s Hospital, London, UK; 2 William Harvey Institute, Queen Mary of London University, London, UK; 3 Department of Thoracic Surgery, Royal Victoria Hospital, Belfast, UK; 4 Chair of Women in Cardiothoracic Surgery, Society of Cardiothoracic Surgery of Great Britain and Ireland (SCTS), London, UK; 5 Department of Cardiothoracic Surgery, University Hospital of Wales, Cardiff, UK; 6 Chair of Women in Cardiothoracic Surgery, European Association of Cardiothoracic Surgery (EACTS), Windsor, UK

**Keywords:** Cardiothoracic surgery, Women in cardiothoracic surgery, Gender, Education, Equality, Diversity

## Abstract

Cardiothoracic surgery is one of the most gender-imbalanced specialties, with women representing less than 10% of the workforce worldwide. Despite a notable increase in recruitment and certification into the specialty in recent years, the disproportion is still notable and there is no expectation for that gap to be reduced in the next decades. Women in cardiothoracic surgery experience unique challenges such as inherited bias, discrimination, sexual misconduct, and extreme imbalance in work-life balance, especially around pregnancy and childcare. These challenges are not only important during recruitment into the specialty and training but continue throughout the whole surgical career. Despite recent efforts from surgical societies worldwide to augment women in cardiothoracic visibility and support, a lot of work is needed to facilitate the path for future generations.

## Introduction

The cardiothoracic surgery (CTS) workforce, and especially cardiac over thoracic surgery, is heavily underrepresented of female cardiac surgeons. Despite gender parity among medical students, the recruitment of females into cardiothoracic training programs is lower than in other surgical specialties. These inequalities extend throughout the cardiothoracic surgical career, with sex-based differences in numbers practicing cardiac surgery, opportunity for promotions, leadership positions, and in some cases remuneration.

Despite the fact that the number of women in CTS (WICTS) has increased significantly in the last decade, there is still a clear imbalance, not only with respect to males in CTS, but also with respect to female representation in other specialties (both medical and surgical).

One of the main reasons to support gender equity in the specialty is the predicted worldwide shortage of CTS specialists in the coming decades and the need to fulfill the increasing numbers of our aging population. Recruiting qualified CTS of all genders is very important and women represent a large resource, given the current gender distribution among medical students [1].

Challenges to attract females to CTS can be due to the following factors, which might differ along the entry point and career pathway:

- Recruitment into the specialty: implicit bias and stereotypes, negative experiences with surgery, and lack of mentorship availability [2].
- Training programs: gender discrimination, lower career satisfaction, imposter syndrome, more disturbed work-life balance, and shortage of mentorship and sponsorship [2, 3].
- Professional career: disparities in allocations of research funding, authorship roles, leadership positions, and pay equity [4–6].

In this narrative review, we analyze most of these areas, aiming to highlight not only the inequities but also to encourage WICTS to overcome these challenges, finding the right inspiration and allies.

## Current representation of WICTS in different healthcare systems

Representation of WICTS has steadily continued to grow worldwide; however, women remain underrepresented compared to their male counterparts. According to the Cardiothoracic Surgery (CTS) Network (CTSNet), women constituted 8% of nearly 15,000 surgeons practicing CTS worldwide. Women currently account for < 4% of all American Board Certified surgeons and < 5% of all practicing CTS in the United States (US) [2] (Table 1).

**Table 1:** Percentage of representation of women in cardiothoracic surgery (*CTS*) in different geographical locations

	Percentage of WICTS
CTS Network (Worldwide)	8%
EACTS (Europe)	16%
United States	12%
Canada	10%
South America	4.6%
Great Britain and Ireland	9%
Spain	28%
Germany	25%
Portugal	16%
Italy	9%
Scandinavia	12%

Data obtained from Cardiothoracic Surgery (*CTS*) Network, European Association of Cardiothoracic Surgery (*EACTS*), Society of Cardiothoracic Surgery for Great Britain and Ireland (*SCTS*), and Spanish Cardiac and Endovascular Surgery Society (*SECCE*), and Appendix 1 of [6]

The European Association of Cardiothoracic Surgery (EACTS) has 4317 members from 121 countries, with 712 female members constituting 16%. Germany, the United Kingdom (UK) and Italy have the highest female membership in EACTS, with 25 of the 121 countries having no female members (Table 1).

In the UK, there are 59 females practicing CTS among the 40 units in the country, which constitutes barely 9.3% of the national CTS workforce (Table 1).

According to demographic data from the Spanish Society of Cardiovascular Surgery (SECCE), there is a high presence of WICTS in Spain, with 53% of the trainees as well as 28% of cardiac surgery specialists being females (Table 1).

The disparities in WICTS representation vary across world regions, with female representation correlating with national economic development [6], although cultural and political attitudes towards gender equity can independently shape WICTS representation. For instance, in Japan, female surgeons report significant cultural barriers to entry in the specialty, including less family support for their careers and conflicts between domestic duties and surgical demands [7].

Only a small number of WICTS pursue a career in adult cardiac surgery compared to their male counterparts. Most female board-certified CTS dedicate their practice to thoracic surgery, congenital surgery, or mixed CTS practice rather than dedicated adult cardiac surgery [2].

From the American Board of Thoracic Surgery (ABTS) data, we know most current adult cardiac surgeons are in their early careers compared with the other CTS subspecialties, with a difference of over 5 years on average [2], therefore indicating a recent growth in the presence of females in adult cardiac surgery.

However, despite the younger population of adult cardiac surgeons, the growth of this subspecialty is less than for CTS as a whole, and even other surgical specialties, which is concerning for the future recruitment of women into adult cardiac surgery.

## Medical students and perceptions of CTS specialty

The number of women entering medical school has equalized or even surpassed that of men (50–60% medical students are females) [2, 6]. Despite 30% of first-year medical students declaring interest in CTS, this seems to dilute over the years of medical school, with only 15% interested to pursue a CTS career in their final year. In the UK, men were 2.5 times more interested than women in pursuing a CTS career [8].

The characteristics cited by medical students as attractive to the specialty are the clinical impact in patient’s life, being an intellectually challenging speciality, and the social image and salary and future opportunities of the specialty. On the other hand, negative factors cited by students were the difficulty of the specialty, the lengthy training and working hours, competition, stress levels, poor working-life balance, and the difficult personalities of the cardiothoracic (CT) surgeons encountered [8, 9].

Interestingly, 30% of the medical students surveyed have had exposure to the specialty via clinical placements or observerships. Regarding female students’ perceptions, 96% (vs. 0% males) viewed surgery as “unfavourable to their gender” [10]. Moreover, male surgeons are significantly less likely than female surgeons to recommend surgery to a female student [1].

## CTS training programs

According to CTSNet, 20% of worldwide CTS residents are females [6]. The disparity between the number of females entering the specialty training and the number of practicing female surgeons poses the question of what happens down the pipeline with regard to retention into the specialty. Women leave residency and fellowship programs more frequently than men. In a European survey, 37% of WICTS considered leaving the survey at some point in their career due to gender discrimination, and 67% recognized having been unfairly treated due to their gender [11].

Although there is an increase in WICTS in the last few years, cardiac surgery has seen the slowest increase in females compared to other surgical specialties, and it is not predicted to reach gender parity any time soon [6].

## Implicit bias and stereotypes

Bias (implicit or otherwise) is well-documented in contributing to unequal opportunities and lack of career advancement among women and minorities, and this is not unique to medicine and/or CTS, but to all professions [3]. Unconscious bias among managers, lack of female role models, lack of incoming talent, low confidence among women in the field, and lack of work-balance have been the main barriers to hiring and promoting women in industry [1].

The equity/diversity/inclusion movements, recently fuelled by social media, fight unconscious bias mottos such as “prove it again,” referring to the need to show extraordinary evidence of competence — women often must demonstrate evidence of competence, whereas in men, competence is assumed.

The “walking a tightrope” refers to balancing being authoritative and abrasive versus being meek and unqualified [12]. This is a common scenario for WICTS, where females in leading positions are perceived differently than men when conducting the same actions; these contribute to women being more criticized and often feeling less prepared and doubting themselves (the so-called imposter syndrome) when compared to their male colleagues [12].

## Work-life balance

The demanding nature of CTS has undeniable implications for family and relationships. There are several pregnancy-related challenges for female surgeons, including negative judgments of competency and pressure to schedule pregnancies around training [13]. As high as 44 to 65% of WICTS reported their careers impacted in their family planning decisions [11, 14]. Women face more professional disadvantages upon starting a family due to the bad perception of taking long maternity leave, especially during the training programs.

A survey conducted by ABTS among WICTS and their family members revealed that over 60% of partners reported the CT schedule to have a moderate to severe impact on the family, not allowing enough time for personal and family needs. The partners praised the sense of pride, enriched knowledge, opportunities for travelling, financial stability, and access to medical advice [15].

A 14% reported impact on children’s emotional well-being (limited quality time together) and in 15% it had affected their ability to have children. Over 50% of partners took most responsibilities of childcare. The partners praised transmissions of values such as commitment, hard work and discipline, empathy and compassion for others, augmented independence, and ability for self-care [15].

Irregular working hours was associated with job-related stressors contributing to relationship tension and insufficient time together.

## Established CTS practice after qualification

It has been reported that in certain geographical areas, women receive fewer referrals and more significant financial repercussions following medical errors [5, 10, 16].

According to the ABTS, very few individuals have changed specialties, which could suggest high career satisfaction or fear of practice change. The number of specialty changes is lower in those who commit to academic pathways [2].

In a confidential survey conducted by the Association of Surgeons of Great Britain and Ireland, over 50% of women reported experiencing discrimination, while 25% perceived a “glass ceiling” in surgical training [17]. These data suggest that retaining qualified surgeons requires an environment of inclusivity, a sense of belonging, support, and opportunities for advancement.

## Titles and leadership positions

Women face lower advancement career opportunities than men (professorships, leadership positions within CTS societies and editorial boards) [2]. After adjusting for age, years in practice after residency, publications, grants, clinical trial participations, and competence in surgical subspecialties, women are still less likely to be promoted to full professors or even to be invited as ground round lectures, visiting professors, leaders of national organizations, members of grant panels, and/or editorial boards [2].

Women present at as many conferences as male colleagues, but publications written by women have fewer citations than those written by men [4]. Involvement in research is positively correlated with increased success in obtaining an academic or leadership position. Women are less likely to continue research during their careers, despite similar previous research experience, reflecting the imbalance in grant opportunities [5, 14]. However, according to the ABTS, the number of female academic surgeons with positions of leadership is almost double that of female leaders in any of the CTS subspecialties, suggesting that after qualifying, academic practice seems to be a preferred career choice for females [2].

## Salary discrepancy

Despite an advanced cardiac surgery infrastructure and high educational attainment for women, in certain geographical locations there is salary discrepancy based on sex. For example, WICTS in North America (both US and Canada) earn significantly less than their male colleagues — their salaries are reported to be only 70–80% of their male peers, even with equal positions, working hours, and similar operative outcomes and hours [2, 5, 16]. Fortunately, this discrepancy is not common worldwide, and other factors such as nationality and years worked in the national healthcare system may well play a role in those salary discrepancies.

## Mentorship

Mentors influence career decisions regarding which specialty to pursue and how best to enter the field [18]. The impact of mentorship is well-documented: 80% of cardiac surgery residents endorsed their specialty choice in mentorship opportunities [19]. Success of mentorship relationships depends on many factors, involving both parties. From the mentee, it requires initiative to develop the relationship, self-reflection, and willingness to change and provide feedback. From the mentor, it requires active listening, understanding the mentee’s internal motivators, and avoiding conflict of interest with any personal or supervisory roles [18].

Although most CTS trainees have mentors during their residency, it does not necessarily mean that those individuals will be able to provide advice regarding work-life balance, long-term career guidance, or assistance in obtaining a job [18].

WICTS value more the role that their mentors play in providing sponsorship and assistance in networking than their male counterparts do [18] and they report more desire for mentorship by individuals of the same sex [20]. Limited female mentorship has been reported as the most consistent barrier cited by female medical students and young trainees when describing challenges and difficulties in entering training programs or thriving in them. Ahmadiyeh et al. found that having female role models in surgical departments increases the likelihood of female students pursuing surgical careers [21]. Because there are a few WICTS, finding a gender-matched mentor can be a challenge for females, particularly with an emphasis on cardiac surgery as opposed to thoracic surgery, limiting the progress of those interested in the field [19].

With the shortage of visible female mentors, it is paramount to ally males with shared vision and values to tackle issues such as female discrimination, sexual harassment, and improving the workplace environment for females. The authors acknowledge that having same-sex mentorship may have intrinsic bias, and the goal should be to find individuals who can encourage and support the intrinsic needs of their mentees, regardless of their sex or background.

## Bullying and sexual harassment

Bullying is defined as offensive, intimidating, or insulting behavior, and abuse or misuse of power through means that undermine or humiliate someone. In a survey among National Health Service (NHS) staff, 38% reported experiencing one or more types of bullying, and 42% had witnessed others being bullied. Bullying was associated with lower levels of psychological health and reduced satisfaction, as well as higher intention to leave work. In total, 55% of the survey respondents experienced bullying, and 78% witnessed it, yet 67% did not report it [22].

Discrimination is defined as unfair and unequal treatment of somebody based on their race, ethnicity, gender, sexuality, disability, or religious beliefs. A survey reported 52% racist conducts by senior colleagues, 38% suffered discrimination, and 62% witnessed it, although 69% did not report it [23].

Sexual misconducts, including harassment, assault, and rape, are considered criminal acts in society, but surprisingly they still occur more often than thought in surgical environments due to a combination of a deeply hierarchical structure and a gender and power imbalance. In a shocking article published in 2023 from the NHS analyzing data over the last 5 years among the surgical workforce, 63.3% of the female respondents, along with 23.7% of men, reported sexual harassment; specific details were given by 872 episodes of sexual harassment, 81 sexual assaults, and 5 rapes — but only 16% of incidents were formally reported [24]. During surgical training, 24% of the trainees experienced harassment, 30% witnessed it, and 72% did not report it [23].

Results indicate a widespread lack of confidence in the adequacy of regulatory bodies and accountable organizations in handling these issues. This reflects a serious issue within the surgical workforce, with implications for patient safety. Perpetrators of these acts are usually male colleagues and demonstrate a need for power, selecting conducive workspaces where they can dominate their domains as they are often too significant to be called out and removed.

Even tolerance to “banter jokes” is not innocuous, offering subtle means for perpetrators to test boundaries, identify like-minded individuals, and desensitize others [23].

Therefore, we can conclude there is a strong presence of poor behavior in the surgical workforce and that women and men are living a different reality, with females being targets of sexual misconduct at higher rates than men. The predominant male senior workforce, hierarchical structures and high-stress environments, unwanted language, and breaches of personal space have been normalized as acceptable behaviors in these environments, resulting in an unsafe working environment and unsafe space for patients. The main barriers to reporting were the perception that nothing would change (reflecting a lack of confidence in the adequacy of regulatory bodies and accountable organization in handling these issues) and not wanting to be considered a troublemaker.

Exposure to sexual misconduct deeply affects an individual’s psychological responses, altering their assumptions, beliefs, and expectations about themselves and others, and being more likely to suffer burnout, which increases the risk of harm to patients; even individual consequences including depression, self-harm, and suicidal ideation [23].

## Positive attributes inherent to WICTS

Female representation in CTS is important to ensure that women’s health remains an active priority among physicians and researchers. It is well known that cardiovascular disease is the leading cause of death for women in first-world countries, and that women have worse outcomes than men when facing cardiac and/or aorto-vascular surgery [25, 26].

The quality of surgical care improves when patients are treated by a team that is similar to them, as they tend to better understand the patients [27].

Women are more likely to adhere to clinical guidelines and generally are more empathetic communicators, which leads to more forthcoming conversations with patients, and overall, this positively influences patients’ outcomes [28]. Women physicians are also more likely to provide preventative medicine and implicate psychosocial factors into their diagnostic workups [29].

There is no difference when comparing sex regarding surgical outcomes. Moreover, a publication established the association between gender discordance and worse postoperative outcomes for female patients treated by male surgeons. On the other hand, no negative effect for male patients treated by female surgeons has been proven [28]. Potentially, females have better surgical outcomes overall, including reductions in mortality and readmission rates, better risk assessment, diagnostic accuracy, and more positively rated patient encounters [16, 28]. Furthermore, male physicians with greater exposure to female physicians were more effective in treating female patients after myocardial infarction [30].

## Future directions for WICTS

Visibility of WICTS is crucial to inspire the next generation of surgeons. The increasing presence of female groups within all professional surgical societies is particularly effective, having increased the female representation at scientific meetings, both in chairing sessions and as abstract presenters at scientific meetings. Dedicated awards and grants also enhance visibility and recognition of female surgeons.

The EACTS WICTS committee conducted the first breakfast session at the Annual meeting in Lisbon in October 2024, which had a turnout of women surgeons from 14 countries, and the unique challenges faced in different countries regarding training, career progression, leadership roles, pregnancy, work-life balance, and entrenched cultural issues were highlighted. Ethnic women in Western countries face a double jeopardy. The EACTS WICTS has additionally instituted 2 Francis Fontan Leadership Fellowships every year to promote female leadership. Efforts are underway to increase the number of women represented in the scientific fora and address mentorship issues. WICTS will network and consolidate the female membership and aims to provide leadership to suggest policy changes that may improve the work-life balance.

Managers of surgical departments should rethink the traditional volume of work, which is unattractive to the new generations who value their work-life balance, posing a substantial barrier to women, who often have greater home responsibilities than their male counterparts. One solution would be the consideration of more flexible work arrangements with greater autonomy in scheduling surgeries rather than fixed sessions. Also, policies supporting earlier childbearing, support during parental leave, and on-site childcare facilities should be provided widely.

Social media and digital platforms have an important role in our current era and can be exploited to enhance networking of medical students and trainees as well as widen the exposure to same sex mentors, allowing the small number of women leaders to reach larger audiences of juniors [20]. These platforms also allow connecting to peers and maintaining those mentoring interactions, helping to combat feelings of isolation often experienced by women in minorities.

Nevertheless, the authors acknowledge that it is well possible that females are less interested in certain surgical specialties than their male counterparts, but there is no doubt that we see an increasing number of females medical students interested in exploring CTS as a career. It is for that audience and for the current WICTS in training that the sex discrepancies highlighted above should be mitigated soon, ensuring that the increasing interest in our specialty is not discouraged.

## Limitations

It is difficult to extrapolate and analyze data coming from surgical databases as not all the practicing surgeons might be enrolled on them; also, not in all geographical regions is cardiothoracic surgery a united specialty, while in others, surgeons might be practicing both specialties.

## Conclusions

The challenges faced by females aspiring or invested in cardiothoracic surgical careers highlight the need for improving workplace conditions, emphasizing the importance of equity, diversity, and inclusion. Work remains to be done to ensure all potential talent is encouraged and supported.

Gender-balanced healthcare teams and the positive influence of female physicians in their male colleagues ultimately benefit patients’ care.

## Data Availability

Available upon request.
